# A Divergent Paired Electrochemical Process for the Conversion of Furfural Using a Divided‐Cell Flow Microreactor

**DOI:** 10.1002/cssc.202002833

**Published:** 2020-12-22

**Authors:** Yiran Cao, Jasper Knijff, Amin Delparish, Maria Fernanda Neira d'Angelo, Timothy Noёl

**Affiliations:** ^1^ Department of Chemical Engineering and Chemistry Sustainable Process Engineering Eindhoven University of Technology (TU/e) Het Kranenveld (Bldg 14-Helix) 5600 MB Eindhoven (The Netherlands; ^2^ Flow Chemistry Group van't Hoff Institute for Molecular Sciences (HIMS) University of Amsterdam (UvA) Science Park 904 1098 XH Amsterdam (The Netherlands

**Keywords:** divided-cell reactor, electrochemistry, flow chemistry, furfural, paired electrolysis

## Abstract

Furfural is a prominent, non‐petroleum‐based chemical feedstock material, derived from abundantly available hemicellulose. Hence, its derivatization into other useful biobased chemicals is a subject of high interest in contemporary academic and industrial research activities. While most strategies to convert furfural require energy‐intensive reaction routes, the use of electrochemical activation allows to provide a sustainable and green alternative. Herein, a disparate approach for the conversion of furfural is reported based on a divergent paired electrochemical conversion, enabling the simultaneous production of 2(5*H*)‐furanone via an anodic oxidation, and the generation of furfuryl alcohol and/or hydrofuroin via a cathodic reduction. Using water as solvent and NaBr as supporting electrolyte and electron‐mediator, a green and sustainable process was developed, which maximizes productive use of electricity and minimizes byproduct formation.

## Graphical abstract

**One road diverged**: Herein, a divergent paired electrochemical conversion of furfural is described, where the cathodic and anodic reactions are separated by a membrane and are productively used to generate valuable derivatives. This strategy enables the simultaneous production of 2(5*H*)‐furanone via an anodic oxidation and the generation of furfuryl alcohol and/or hydrofuroin via a cathodic reduction.

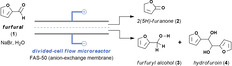

Furfural is one of the most prominent biobased molecules, registered as a top 30 biomass‐derived platform molecule by the US Department of Energy.^[1]^ It is obtained by hydrolysis and dehydration of xylan, which is abundantly available from hemicellulose. Furfural is currently produced on a 300 k Ton per annum scale and about 70 % of its production is carried out in China.^[2]^

The establishment of furfural as a commodity chemical spurs academic and industrial interest to develop new synthetic routes for its further derivatisation into useful chemicals, materials, and biofuels.[^3^, ^4^, ^5^] Amongst the different strategies used to convert furfural (e. g., pyrolysis, gasification, thermo‐catalytic processes), the use of electrochemical activation enables a green and sustainable alternative to these often high‐energy‐demanding processes.[^6^, ^7^, ^8^, ^9^, ^10^] Notably, electrochemistry allows to convert green electricity, derived from wind and solar energy, directly into useful chemicals, and kinetic barriers are overcome by applying a suitable potential over the electrodes. Hence, no additional reagents are required to enable reduction[^11^, ^12^, ^13^, ^14^] and oxidation processes,^[15]^ which can aid to further reduce fossil fuel consumption (e. g., hydrogen is often derived from natural gas via the water‐gas shift reaction).^[16]^

While several useful electrochemical strategies for the conversion of furfural have been developed, the focus so far was almost exclusively on the optimization of a single electrode reaction. However, when both electrode reactions are harmonized to produce value‐added products, a green and sustainable synthesis can be obtained, which maximizes productive use of electricity, minimizes waste generation, and reduces energy consumption.[^17^, ^18^, ^19^, ^20^] Such a coupled process, also called “paired electrolysis”, would be of great added value for the conversion of furfural, where the economical margins are often low.

Herein, we describe such a divergent paired electrochemical conversion of furfural, where the cathodic and anodic reactions are separated by a membrane and are productively used to generate valuable derivatives (Figure 1A). To further increase the utility and scalability of the process,^[21]^ the reactions are carried out in a continuous‐flow electrochemical reactor with a narrow inter‐electrode gap (Figure 1B).[^22^, ^23^, ^24^, ^25^, ^26^] The selectivity can be increased in such reactors due to the large electrode surface‐to‐volume ratio and due to a meticulous control over the residence time and the cell potential. Using such intensified reaction conditions, the reaction time is typically reduced significantly compared to batch cells and, thus, the products are only briefly exposed to the electrochemical conditions, efficiently avoiding undesired follow‐up reactions.[^27^, ^28^, ^29^] In this work, we show that a divergent paired electrochemical flow strategy enables the simultaneous production of 2(5*H*)‐furanone via an anodic oxidation, and the generation of furfuryl alcohol and/or hydrofuroin via a cathodic reduction (Figure 1A). It should be noted that these three biobased compounds derived from furfural have great synthetic and practical value. 2(5*H*)‐furanone can readily be reduced to generate *γ*‐butyrolactone,[^30^, ^31^] a valuable biobased solvent, synthetic intermediate, and monomer for the synthesis of poly(*γ*‐butyrolactone).^[32]^ Furfuryl alcohol is mainly used as a raw material to produce furan‐based foundry resins,^[13]^ while hydrofuroin can be utilized as a jet‐fuel precursor.^[33]^

**Figure 1:**
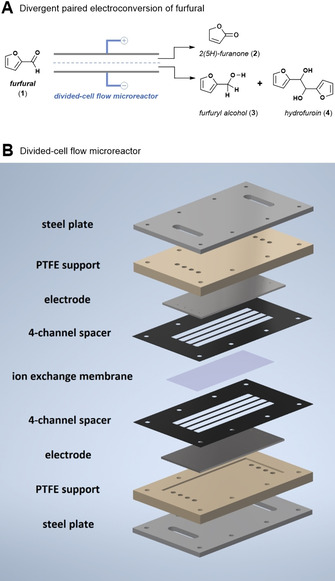
(A) Processes based on a divergent paired electrolysis of furfural allows to obtain useful derivatives of both cathodic and anodic processes simultaneously. (B) Schematic representation of the divided‐cell flow microreactor design using an ion‐exchange membrane to separate the anodic and the cathodic electrolysis half‐cells.

We commenced our investigations by repurposing our original electrochemical flow reactor design (Figure 1B).^[34]^ An ion‐exchange membrane was used to separate the anodic and the cathodic electrolysis half‐cells.^[35]^ Both cation‐ (Nafion XL) and anion‐exchange (Fumasep FAS‐50) membranes^[36]^ were purchased and could be readily sandwiched between two Teflon reaction channel spacers as shown in Figure 1B. The reaction solution was introduced into the two half‐cells of the electrochemical flow reactor using syringe pumps (Fusion 200, Chemyx). The catholyte and anolyte were separately collected and analysed by GC‐FID (FID: flame ionization detector).

Prior to the optimization studies, a voltammogram was recorded to establish the operational windows for the electrolysis of furfural (Figure 2). Two clear plateaus can be distinguished at 2.3–2.5 V and 2.8–3.0 V for both the cation‐ and the anion‐exchange membrane. The current is higher at any given potential for the cation‐exchange membrane, suggesting a lower resistance for ion transport compared to the anion‐exchange membrane.

**Figure 2:**
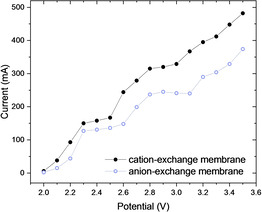
Voltammogram of furfural in the divided‐cell flow microreactor design using an anion‐ or a cation‐exchange membrane.

Based on these results (Figure 2), we decided to use our flow cell in a constant‐potential or potentiostatic operation mode and set the cell potential at either 2.4 or 2.9 V (Table 1; see also the Supporting Information). In our experience, a potentiostatic mode in combination with a continuous‐flow operation allows to obtain high selectivities for a targeted compound without the need to extend the reaction times.[^29^, ^37^, ^38^] Extended reaction times, observed with potentiostatic electrochemical transformations, is an often encountered problem in batch electrolysis as the current decreases with increasing conversions.^[39]^ However, in flow, high substrate concentrations are encountered at the entrance of the reactor and low concentrations at the exit, leading to an average current density over the entire reactor length. This phenomenon in combination with the high electrode surface‐to‐volume ratio results in significantly reduced reaction times in flow.^[22]^ An aqueous mixture of furfural and NaBr, as supporting electrolyte and potentially as anodic electron mediator^[40]^ facilitating the oxidation process,[^41^, ^42^] was infused into the reactor. The two half‐cells are separated by a Fumasep FAS‐50 anion‐exchange membrane and graphite was initially selected as anode and lead as the cathode, which has a high hydrogen overpotential (Table 1, entry 1).^[43]^ Suppression of the hydrogen evolution reaction is important to ensure high faradaic efficiencies[^44^, ^45^] and to avoid gas formation which leads to a higher ohmic drop.^[46]^ As can be seen from Table 1, within 5 min residence/reaction time, good yields are obtained for 2(5*H*)‐furanone at the anodic half‐cell and furfuryl alcohol and hydrofuroin at the cathodic half‐cell. At a cell potential of 2.4 V, a higher selectivity is observed for furfuryl alcohol at the cathode (Table 1, entry 1). A higher yield for both 2(5*H*)‐furanone and hydrofuroin are observed at a cell potential of 2.9 V, respectively 77 and 71 % (Table 1, entry 1). This result demonstrates that small changes in cell potential allow to fine‐tune the selectivity of the electrochemical redox process. Other electrode materials, such as Ni, 316 L stainless steel, Monel 400, or copper, showed a reduced efficacy to generate the targeted compounds (Table 1, entries 2–9).

**Table 1:** Screening of electrode materials for the paired electrolysis of furfural.^[a]^

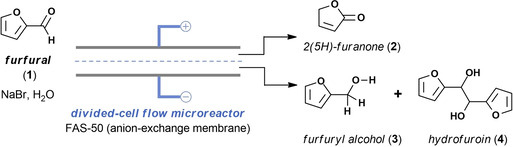								
Entry	Anode	Cathode	Yield 2^[b]^ [%]		Yield 3^[b]^ [%]		Yield 4^[b]^ [%]	
			2.4 V	2.9 V	2.4 V	2.9 V	2.4 V	2.9 V
1	G	Pb	46	77	58	20	29	71
2	Ni	Pb	trace	11	54	17	18	46
3	316L	Pb	trace	10	45	10	9	32
4	Monel 400	Pb	14	23	60	26	12	55
5	G	Cu	40	73	56	24	25	64
6	G	Monel 400	36	70	47	9	23	62
7	G	Ni	32	65	51	17	17	49
8	G	G	32	69	28	trace	20	20
9	G	316L	27	49	34	trace	trace	33

[a] Reaction conditions: 0.1 m furfural, 0.1 m NaBr, H_2_O, 5 min residence time, Fumasep FAS‐50 as an anion exchange membrane. [b] GC‐yield using GC‐FID with internal standard (toluene).

An investigation of the yield and selectivity in function of the residence/reaction time was carried out for both the cation‐ and the anion‐exchange membrane configuration (Figure 3). For the anodic half‐cell (Figure 3A), the highest yield for 2(5*H*)‐furanone (77 % GC yield) is observed at 5 min for the anion‐exchange membrane after which it reaches a plateau. At 5 min residence/reaction time, the yield at the cation‐exchange membrane is only 62 % for the targeted compound (Figure 3A). Interestingly, at the cathodic half‐cell, better results are obtained with the cation‐exchange membrane for the production of furfuryl alcohol and hydrofuroin. However, the difference in performance between the two membranes is less pronounced than for the anodic half‐cell; for example, at 5 min 75 % hydrofuroin and 14 % furfuryl alcohol is obtained for the cation‐exchange membrane versus 67 % hydrofuroin and 26 % furfuryl alcohol for the anion‐exchange membrane. Hence, we selected the anion‐exchange membrane for our further studies.

**Figure 3:**
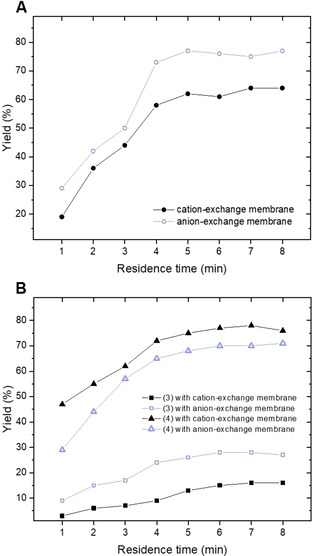
Residence time screening for the (A) anodic half‐cell yielding 2(5H)‐furanone (2), and (B) cathodic half‐cell yielding furfuryl alcohol (3) and hydrofuroin (4). Reaction conditions: 0.1 m furfural, 0.1 m NaBr, H_2_O, graphite anode|lead cathode, cell potential 2.9 V. Yields obtained with GC‐FID and internal standard calibration (toluene).

Next, we investigated the effect of various halide sources on the reaction outcome (Table 2). The best results were obtained with bromide salts (Table 2, entry 1 vs. entry 2). It is known that bromide ions enable mild and indirect anodic oxidations via either hypobromite (BrO^−^) or bromonium (Br^+^) intermediates.[^47^, ^48^, ^49^, ^50^] Other alkali bromide sources were also effective, but the best results were obtained with cheap and abundantly available NaBr (Table 2, entries 1, 3–7). Addition of small quantities (10 vol %) of organic solvents did not lead to any significant improvement and was even less effective in the cases of methanol and THF (Table 2, entries 8–10). Interestingly, in the absence of a membrane, a much‐reduced reaction efficiency was noticed (Table 2, entry 11); this observation demonstrates that the presence of a suitable membrane to separate the two half reactions is crucial to obtain high yields and selectivities.

**Table 2:** Influence of halide sources, solvent, and control experiment for the paired electrolysis of furfural.^[a]^

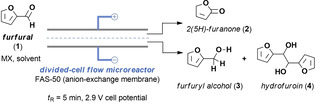					
Entry	MX	Solvent	Yield 2^[b]^ [%]	Yield 3^[b]^ [%]	Yield 4^[b]^ [%]
1	NaBr	H_2_O	77	20	71
2	NaCl	H_2_O	trace	trace	trace
3	LiBr	H_2_O	64	10	32
4	KBr	H_2_O	59	26	55
5	CsBr	H_2_O	61	25	67
6	MnBr_2_	H_2_O	15	7	27
7	TBAB	H_2_O	38	24	29
8	NaBr	H_2_O+CH_3_CN^[c]^	77	27	66
9	NaBr	H_2_O+CH_3_OH^[c]^	58	33	41
10	NaBr	H_2_O+THF^[c]^	52	12	22
11^[d]^	NaBr	H_2_O	trace	16	11

[a] Reaction conditions: 0.1 m furfural, 0.1 m MX, graphite anode**|**lead cathode, residence time 5 min, cell potential 2.9 V, full conversion with anion‐exchange membrane [b] Yields obtained with GC‐FID and internal standard calibration (toluene). [c] The solvent consists of 90 vol % H_2_O and 10 vol % organic solvent. [d] The reaction was done with the same conditions as Entry 1 but in an undivided electrochemical flow cell.

In order to scale up this paired electrolysis of furfural, we wondered if it was possible to increase the concentration of the starting material without compromising the yield and the selectivity (Figure 4). A higher concentration would result in a higher throughput whilst keeping the residence time constant. Indeed, the concentration could be increased up to 0.6 m furfural without reducing the efficacy of the electrochemical process. Higher concentrations were not possible due to the limited solubility of furfural in water.

**Figure 4:**
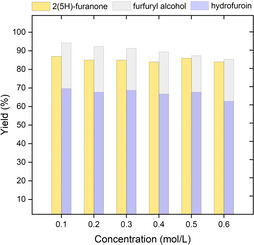
Concentration effect on the divergent paired electrolysis of furfural. Reaction conditions: *x* m furfural, 0.1 m NaBr, H_2_O, 5 min residence time, Fumasep FAS‐50 as an anion exchange membrane. [b] GC‐yield using GC‐FID with internal standard (toluene).

Based on the experimental observations, a plausible mechanism is suggested in Figure 5. In the anodic half‐cell, the C_5_ chemical furfural is converted into the C_4_ building block 2(5*H*)‐furanone in excellent yield. Based on recent work from Han and co‐workers,^[51]^ we suggest that a hydroxyl‐radical‐induced C−C bond cleavage generates the corresponding 2‐hydroxyfuran and formic acid. Subsequent isomerization of 2‐hydroxyfuran generates the observed 2(5*H*)‐furanone. In the cathodic half‐cell, furfural is reduced to generate the corresponding radical.^[44]^ These radicals can dimerize to yield the C_10_ compound, hydrofuroin. Competitively, the radical can consume another electron to generate furfuryl alcohol.

**Figure 5:**
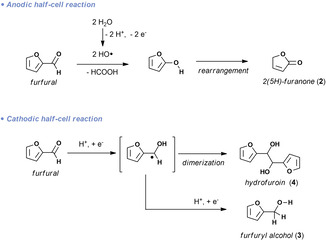
Proposed mechanism for the divergent paired electrolysis of furfura

We have developed a divergent, paired continuous‐flow electrolysis of furfural yielding useful bio‐based chemicals; this includes 2(5*H*)‐furanone via an anodic oxidation, and furfuryl alcohol and hydrofuroin at the cathodic half‐cells. We have shown that it is key to separate the two half‐cell reactions from each other with a membrane to obtain high yields and selectivities. Interestingly, the reactions can be carried out in water as a green solvent and only require NaBr as supporting electrolyte and electron‐mediator. We believe that this paired electrochemical process to convert furfural into useful bio‐based derivatives will be of great added value from the vantage point of an increased productive use of electricity, and a reduction of waste generation and energy consumption.

## Conflict of interest

The authors declare no conflict of interest.

## Supporting information

As a service to our authors and readers, this journal provides supporting information supplied by the authors. Such materials are peer reviewed and may be re‐organized for online delivery, but are not copy‐edited or typeset. Technical support issues arising from supporting information (other than missing files) should be addressed to the authors.

Supplementary
