# Supplementary material for: A Divergent Paired Electrochemical Process for the Conversion of Furfural Using a Divided‐Cell Flow Microreactor
Source: ChemSusChem. 2020 Dec 22;14(2):590–4. doi: 10.1002/cssc.202002833 (PMC7898665; doi:10.1002/cssc.202002833)
Supplement: Supplementary file 1 — Supplementary [file CSSC-14-590-s001.pdf]

ChemSusChem

Supporting Information

A Divergent Paired Electrochemical Process for the Conversion of Furfural Using a Divided-Cell Flow Microreactor

Yiran Cao, Jasper Knijff, Amin Delparish, Maria Fernanda Neira d'Angelo, and Timothy Noël*© 2020 The Authors. ChemSusChem published by Wiley-VCH GmbH. This is an open access article under the terms of the Creative Commons Attribution License, which permits use, distribution and reproduction in any medium, provided the original work is properly cited.

Contents

1. Electrochemical devices S2
2. Pretreatment and cleaning S8
  2.1 Electrode cleaning S8
  2.2 Ion exchange membrane pretreatment S8
3. Procedures S9
  3.1 Operation steps S9
  3.2 Half-reaction optimization S9
4. Characterization data S11
  4.1 NMR spectra S11
  4.2 GC spectra S13
5. Faradaic efficiency S14
6. References S14

1. Electrochemical devices

The flow electrochemical reactor was manufactured by the Equipment and Prototyping Center at Eindhoven University of Technology. The reinforcement part was made of stainless steel (SAE international type 316L), which offered great axial stress with the help of eight pair of bolted joints (bolts with lock washers) distributed on the four edges and fastened by super flangeless fittings. The bolted joints guaranteed the packed structure and provided gas tightness of the reactive part in the cell. The container part was made of polytetrafluoroethylene (PTFE) which offers great chemical resistance and appropriate elastic modulus (proper stiffness for mechanical strength, suitable elastic deformation for sealing the reactor). The spacer part was made of PTFE (0.25 mm thickness) that was treated as insulator to partition the channels in four reactive chambers.

The electrode was made of either stainless steel (316L), copper (Cu-DHP), graphite (AC-K800), nickel (201) and lead (L50049) plates.

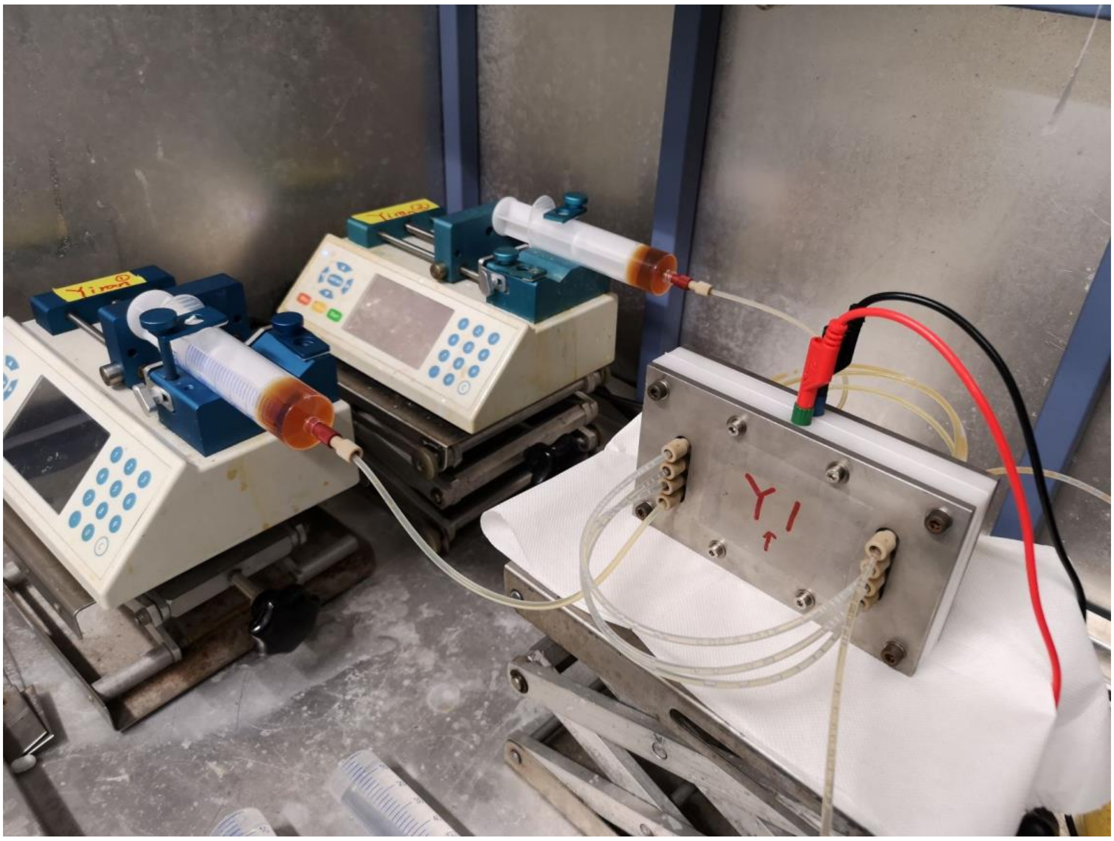

Figure S1. Overview of the electrochemical setup.

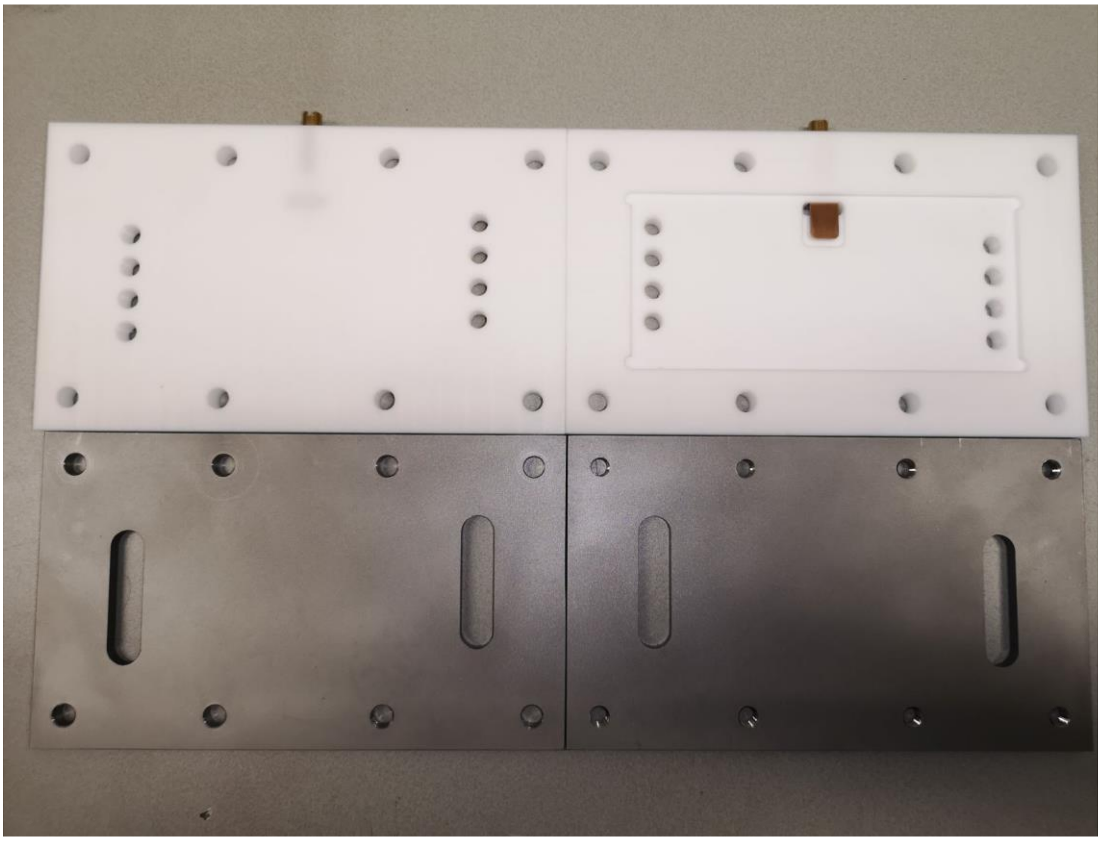

Figure S2. Supporting framework of the electrochemical reactor.

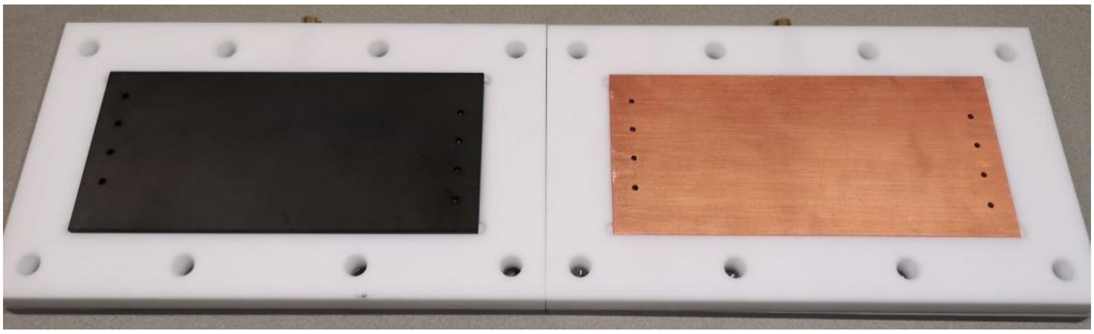

Figure S3. Anodic/Cathodic cell with fitted graphite/copper electrode.

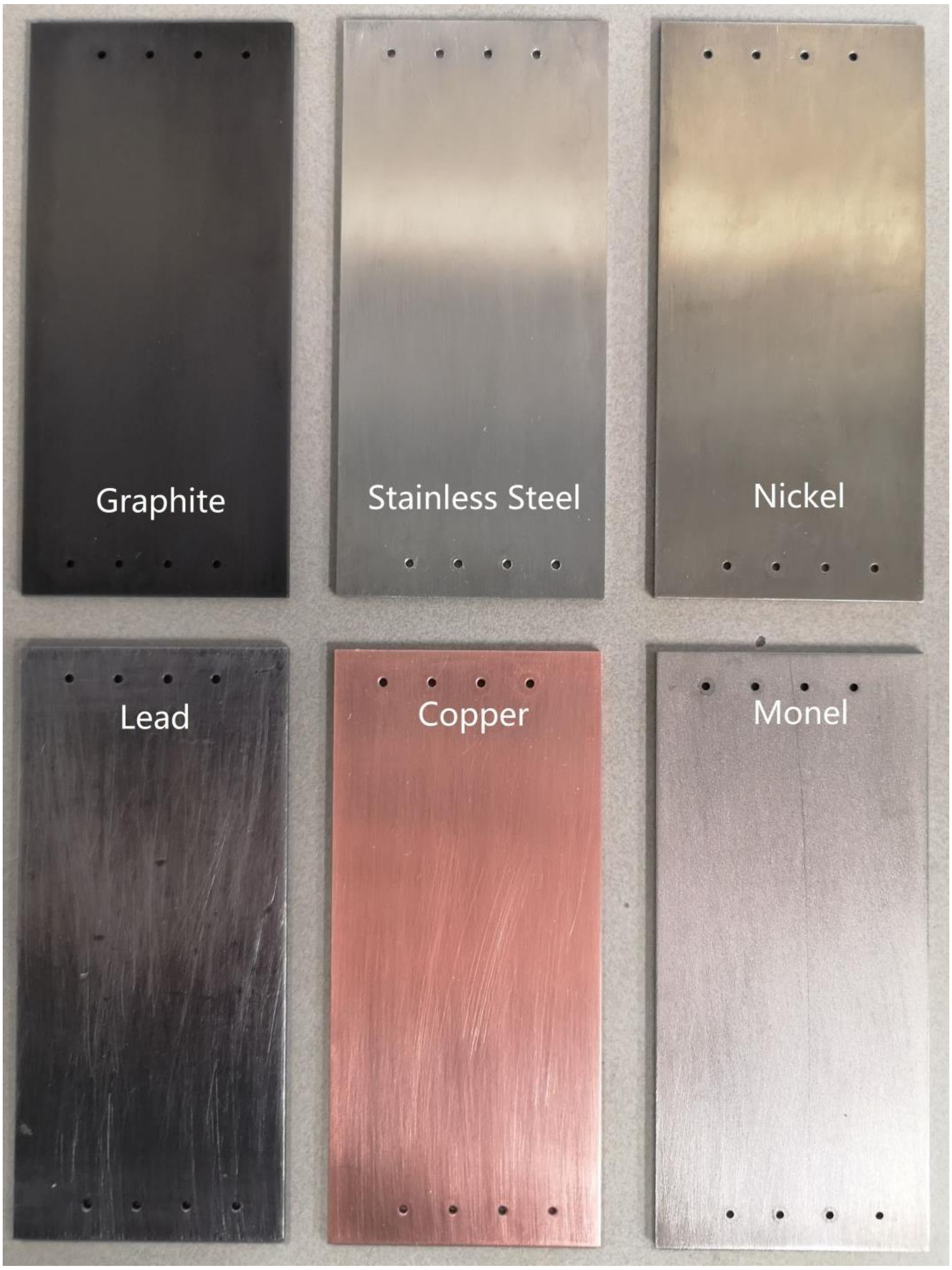

Figure S4. Overview of all the electrodes used in the current study.

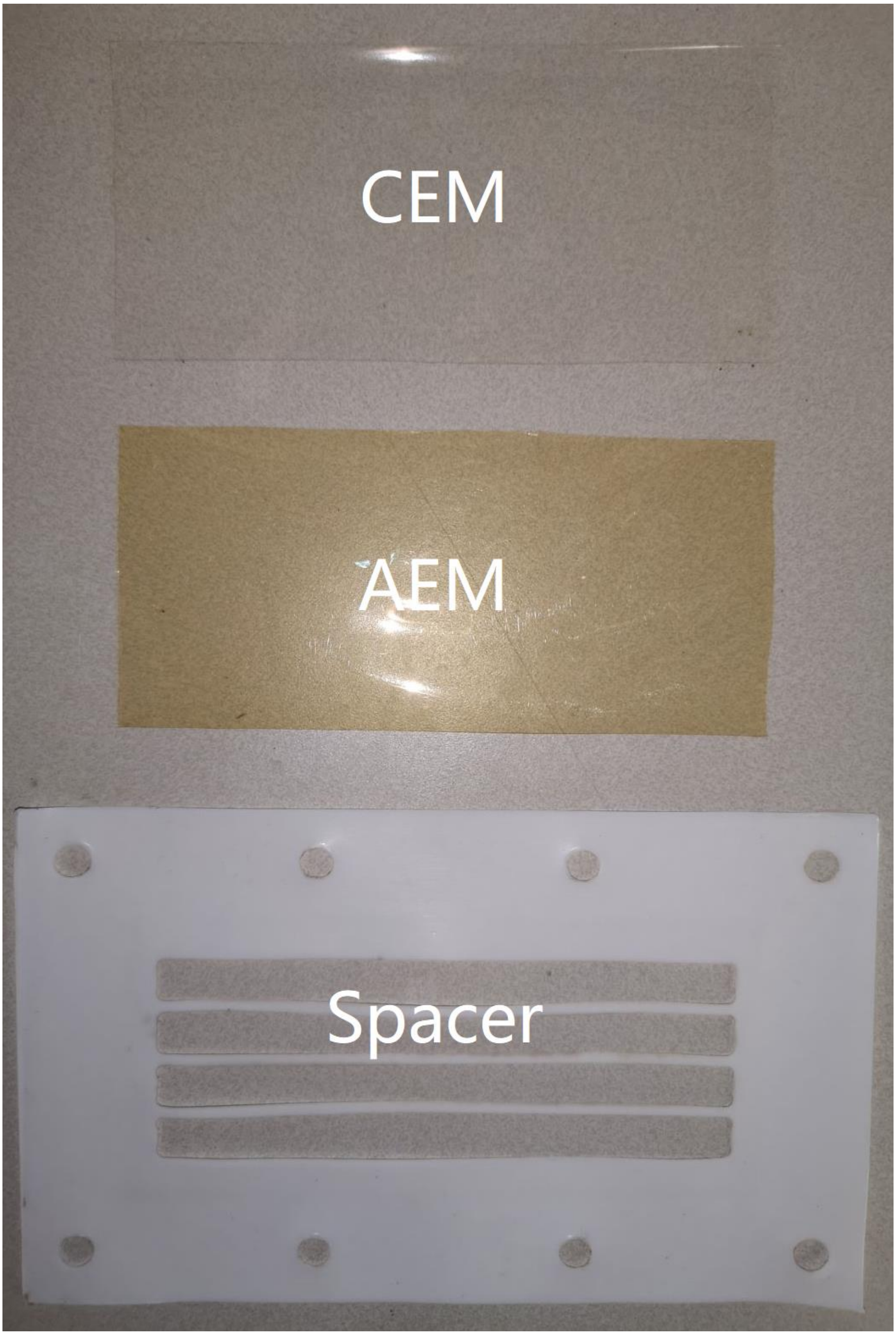

Figure S5. Pictures of the cation exchange membrane, anion exchange membrane and the reaction channel spacer.

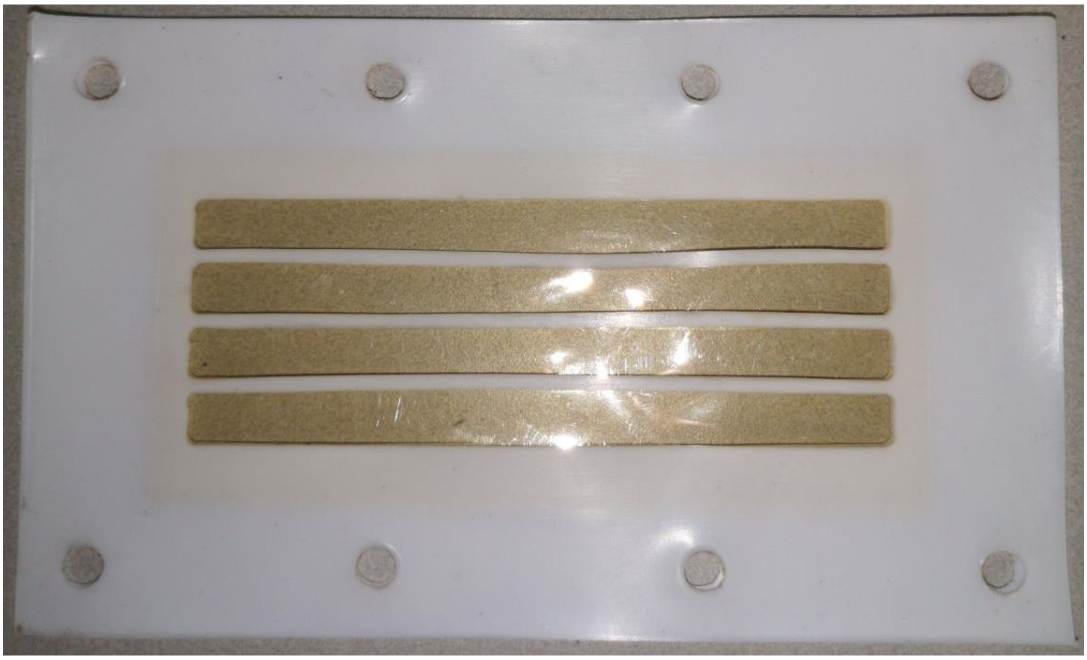

Figure S6. Membrane sandwiched between two reactor spacers.

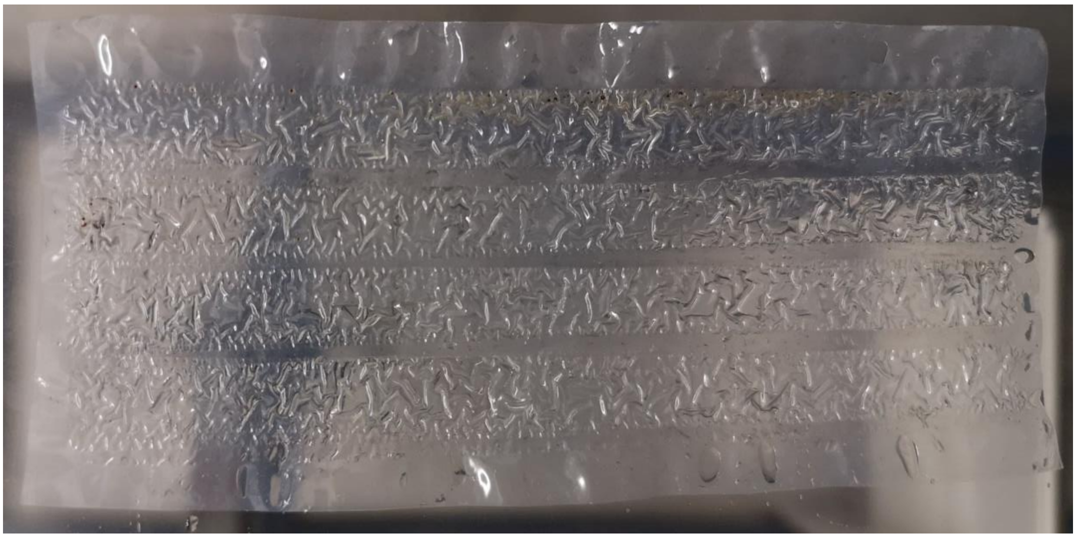

Figure S7. Used membrane with ion exchange channels clearly visible.

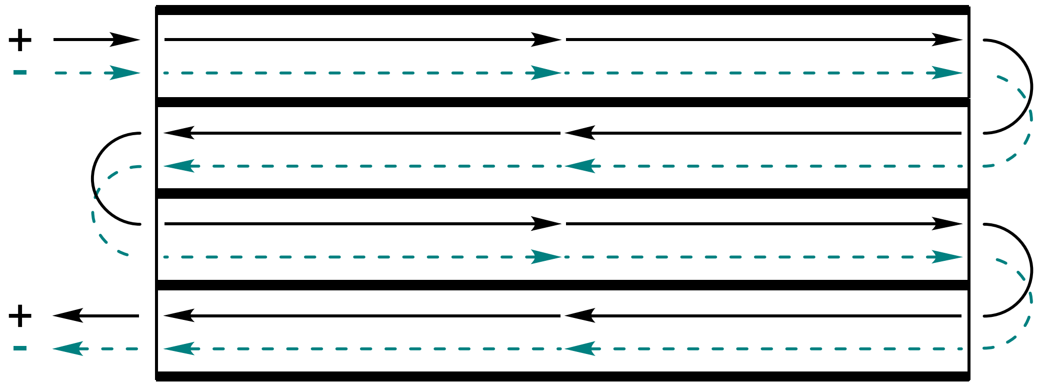

Figure S8. Cocurrent (parallel) flow profile of where the four channels are placed in series in the divided-cell reactor.

2. Pretreatment and cleaning

2.1 Electrode cleaning:[1]

1. Wash the electrodes successively with 1 M HCl and acetonitrile to get rid of any inorganic and organic residuals.
2. Scrub and polish the surface with fine emery paper.
3. Wash the electrodes using 18.2 MΩ H_2O
4. Dry the surface of electrodes.

2.2 Ion exchange membrane pretreatment:[2]

The cation-exchange membrane was placed in a bath of 3% H_2O_2 solution and heated to 80 °C for 1 h under magnetic stirring to remove organic impurities. Then, the membrane was rinsed in de-ionized water and placed in a de-ionized water bath at 100

°C for 2 h under magnetic stirring. Next, the membrane was placed in a bath of 0.5 M H_2SO_4 at 80 °C for 1 h under magnetic stirring. Finally, the membrane was rinsed in de-ionized water at 80 °C and stored in de-ionized water.

The anion-exchange membrane was rinsed in a NaCl solution (0.5 M NaCl solution at T = 25 °C for 24 hours) to remove any additive from the membrane. Place the membrane sample between stabilizing meshes / spacers in order to avoid curling.

3. Procedures

3.1 Operation steps

Stock solutions of furfural and sodium bromide were dissolved in H_2O (18.2 MΩ.cm). The solution was taken up in a syringe and the syringes were mounted on a syringe pump. The solutions were introduced into the electrochemical reactor at the appropriate flow rate. Upon exiting the reactor, the anodic and cathodic reaction mixture was gathered and extracted three times with dichloromethane, dried with anhydrous magnesium sulfate, and concentrated under reduced pressure for further use.

3.2 Half-reaction optimization

As shown in the Table S1 and S2, the yield of the product was stable when given enough electrolyte (0.1 M) while showcasing a great enhancement compared to the paired-electrolysis scenario, from 77% to 88% owing to the fact that hydrogen evolution replaced furfural reduction as the cathodic reaction.

The yield in function of the applied cell potential are shown in Figure S9. The yield of furfuryl alcohol, hydrofuroin and 2(5H)-furanone were compared between the anion-exchange membrane and the cation-exchange membrane, with hydrogen evolution or oxygen evolution in the other half-cell. Furfural alcohol was the predominant product between 2.3 – 2.5 V cell voltage; while 2(5H)-furanone and HF were the main products at the 2.8 – 3.0 V plateau, enabling a high total selectivity for either oxidative/reductive transformation.

Table S1. Anodic reaction optimization. [a]

Channel	Anolyte	Channel	Catholyte	Yield 2 (%)[b]
1a	FUR+0.05 M NaBr	1c	0.05 M NaBr	73
2a	FUR+0.1 M NaBr	2c	0.1 M NaBr	88
3a	FUR+0.2 M NaBr	3c	0.2 M NaBr	87
4a	FUR+0.5 M NaBr	4c	0.5 M NaBr	85

[a] Reaction conditions: 0.1 M furfural, H_2O solvent, graphite anode, lead cathode, residence time 5 min, cell potential 2.9 V with anion exchange membrane. GC-yield was tested by GC-FID with internal standard (toluene). [b] GC yield of 2(5H)-furanone.

Table S2. Cathodic reaction optimization. [a]

Channel	Anolyte	Channel	Catholyte	Yield 3 (%)[b]	Yield 4 (%)[c]
1a	0.05 M NaBr	1c	FUR+0.05 M NaBr	55	55
2a	0.1 M NaBr	2c	FUR+0.1 M NaBr	67	67
3a	0.2 M NaBr	3c	FUR+0.2 M NaBr	69	69
4a	0.5 M NaBr	4c	FUR+0.5 M NaBr	70	70

[a] Reaction conditions: 0.1 M furfural, H_2O solvent, graphite anode, lead cathode, residence time 5 min, cell potential 2.9 V with anion exchange membrane. GC-yield was tested by GC-FID with internal standard (toluene). [b] GC yield of furfuryl alcohol. [c] GC yield of hydrofuroin.

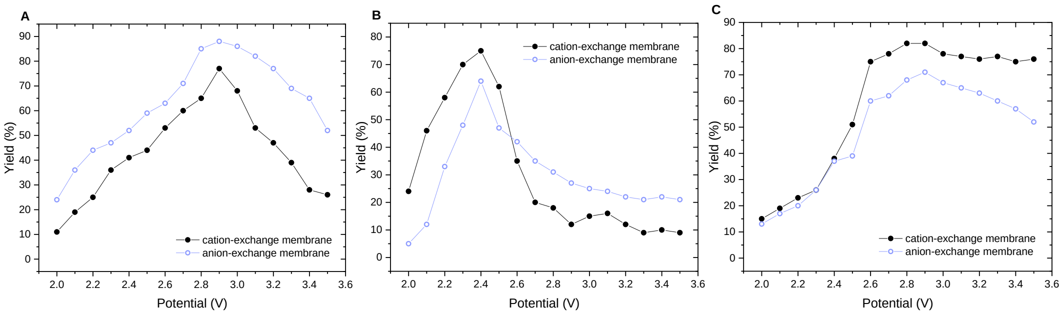

Figure S9. Half-reaction potential screening of (A) 2(5H)-furanone. (B) furfuryl alcohol. (C) hydrofuroin. Reaction conditions: 0.1 M furfural, 0.1 M NaBr, H_2O, graphite anode | lead cathode, 5 min residence time. Yields obtained with GC-FID and internal standard calibration (toluene).

4. Characterization data

4.1 NMR spectra

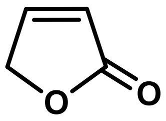

2(5H)-Furanone:[3] Following the procedure 3.1, obtained at 2.9 V for 5 min. Purified and concentrated under reduced pressure to give a light yellow oil.

^{1}H NMR (400 MHz, $CDCl_3$) δ 7.58 (dt, *J* = 5.8, 1.7 Hz, 1H), 6.20 – 6.12 (m, 1H), 4.90 (q, *J* = 1.8 Hz, 2H).

^{13}C NMR (101 MHz, $CDCl_3$) δ 173.77, 152.89 (d, J = 2.8 Hz), 121.72 (d, J = 1.8 Hz), 72.23.

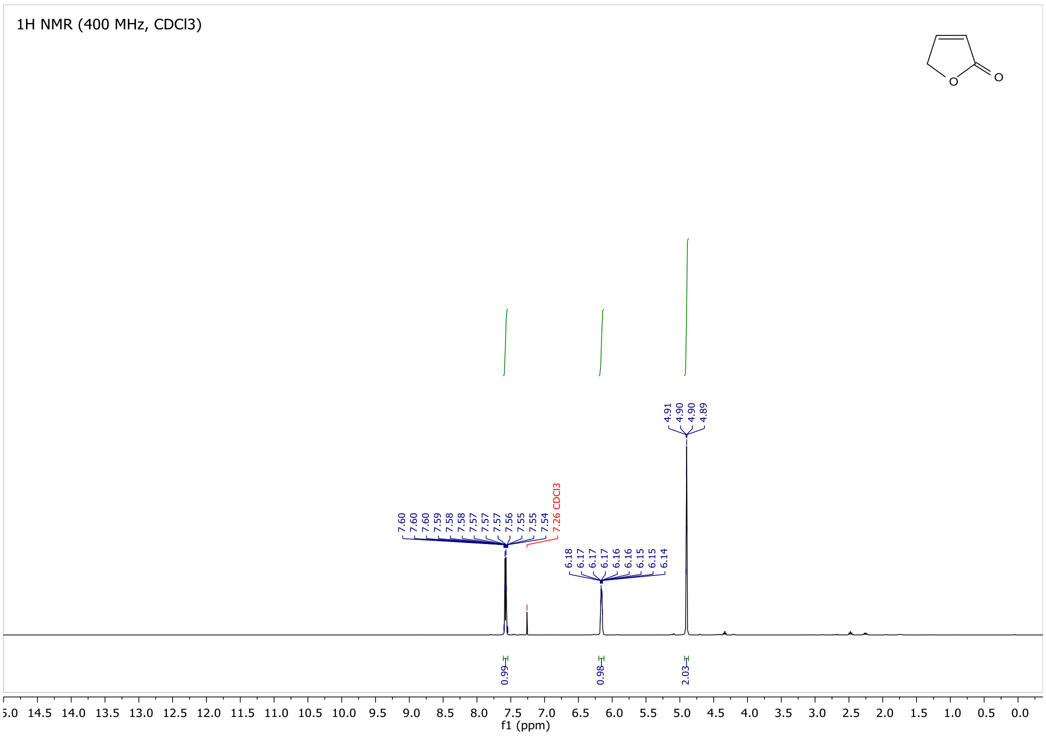

Figure S11. ^{1}H NMR spectra of 2(5H)-furanone.

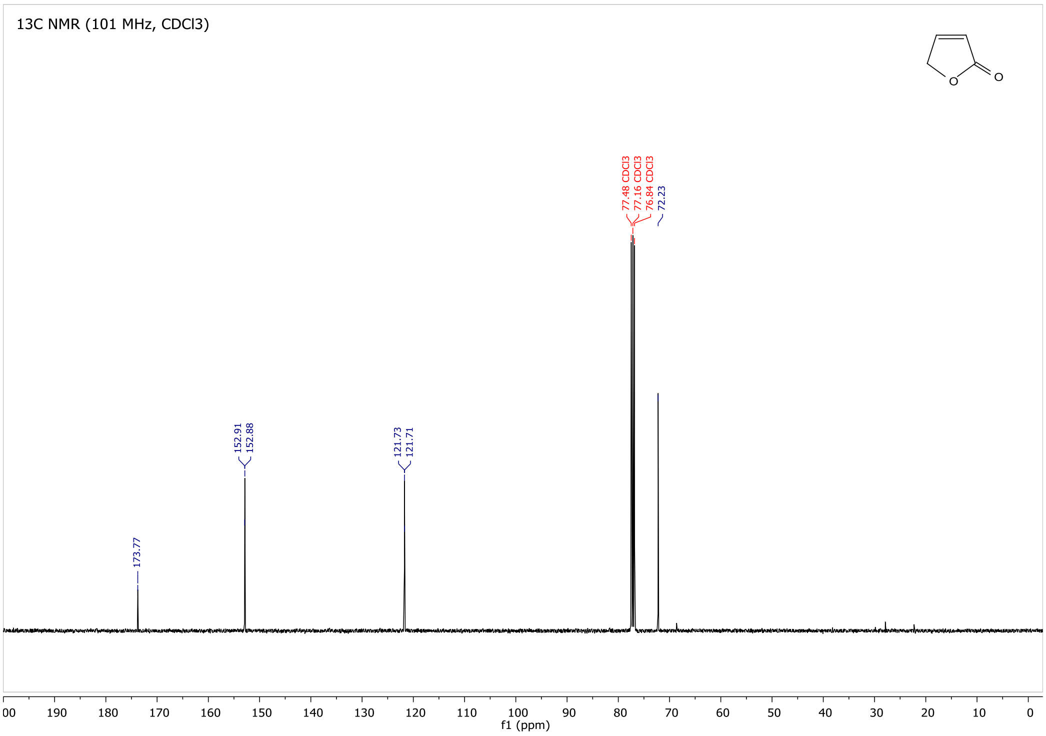

Figure S12. ^{13}C NMR spectra of 2(5H)-furanone.

4.2 GC spectra

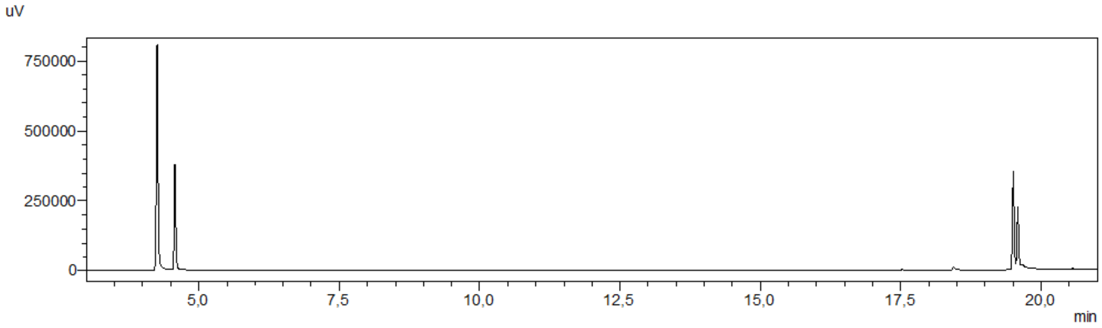

Figure S13. Standard sample of furfural (t = 4.25 min), furfuryl alcohol (t = 4.60 min) and hydrofuroin (t = 19.50 & 19.60 min).

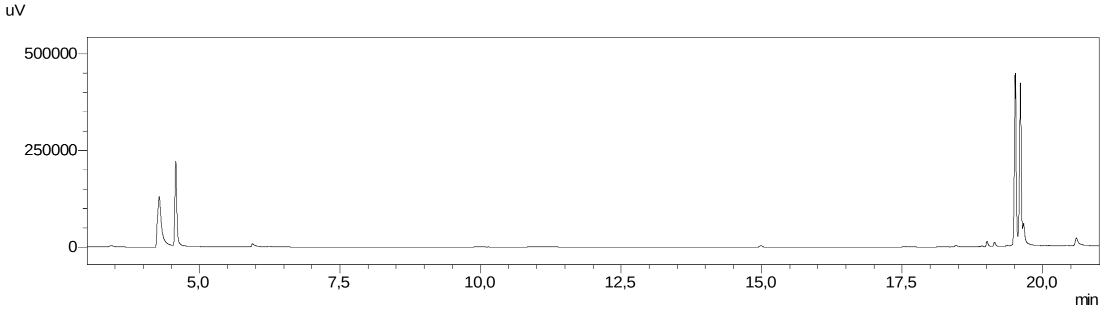

Figure S14. Cathodic reaction mixture, according to the reaction conditions in table 1, entry 1 at 2.9 V cell potential.

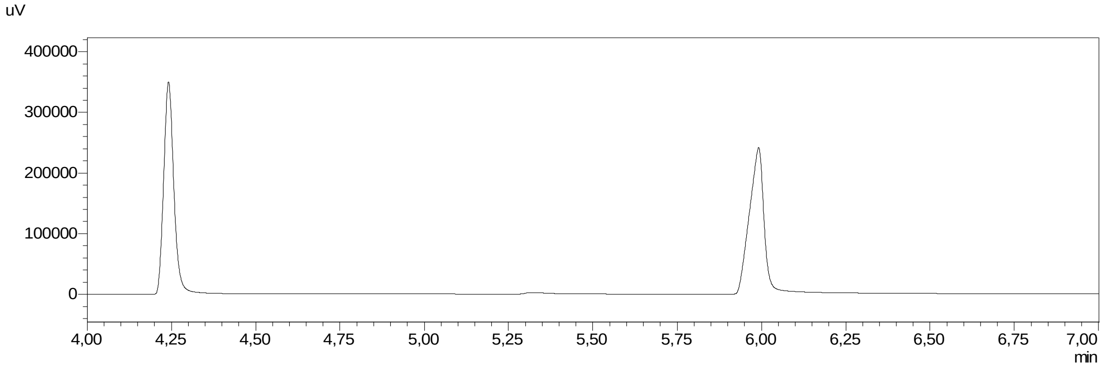

Figure S15. Standard sample of furfural (t = 4.25 min) and 2(5H)-furanone (t = 6.00 min).

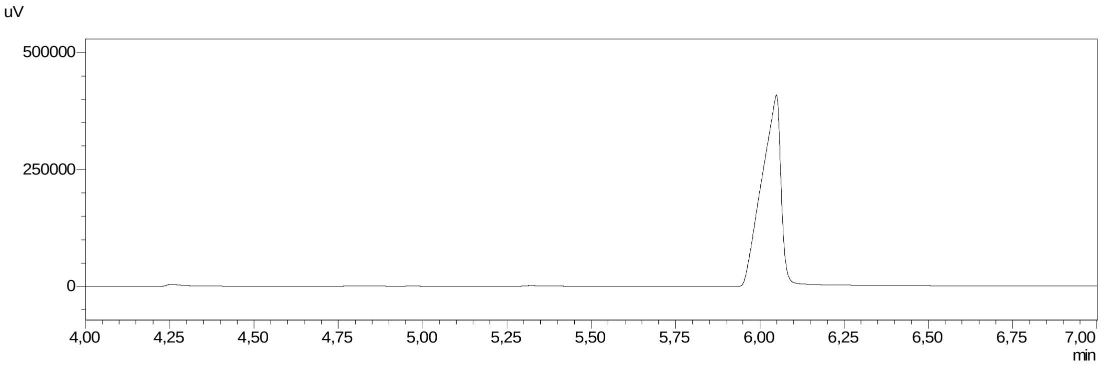

Figure S16. Anodic reaction mixture, according to the reaction conditions in table 1, entry 1 at 2.9 V cell potential.

5. Faradaic efficiency

For the paired electrochemical oxidation of furfural to 2(5H)-furanone

$V_{reaction} = 870\ \mu L$

$n_{substrate} = 2\ mmol$

$\tau_r = 300\ s$

$I_{flow} = 251\ mA$

$F_{flow} = 174\ \mu L * min^{-1}$

$Y_{BNL} = 77\ \%$

$$\eta_{BNL} = \frac{n_{exp}}{n_{theo}} * 100\% = \frac{1.46\ mmol}{2.00\ mmol} * 100\% = 73\ \%$$

For the paired electrochemical reduction of furfural to hydrofuroin

$V_{reaction} = 870\ \mu L$

$n_{substrate} = 2\ mmol$

$\tau_r = 300\ s$

$I_{flow} = 251\ mA$

$F_{flow} = 174\ \mu L * min^{-1}$

$Y_{HF} = 71\ \%$

$$\eta_{HF} = \frac{n_{exp}}{n_{theo}} * 100\% = \frac{0.67\ mmol}{1.00\ mmol} * 100\% = 67\ \%$$

6. References

(1) Hall, L. C. Cyclic Voltammetric Study of Copper Electrode Pretreatment for Metal Migration and Corrosion Rates. *J. Electrochem. Soc.* **1987**, *134* (8), 1902.

(2) Chadderdon, X. H.; Chadderdon, D. J.; Matthiesen, J. E.; Qiu, Y.; Carraher, J. M.; Tessonnier, J.-P.; Li, W. Mechanisms of Furfural Reduction on Metal Electrodes: Distinguishing Pathways for Selective Hydrogenation of Bioderived Oxygenates. *J. Am. Chem. Soc.* **2017**, *139* (40), 14120–14128.

(3) Kortet, S.; Claraz, A.; Pihko, P. M. Catalytic Enantioselective Total Synthesis of (+)–Lycoperdic Acid. *Org. Lett.* **2020**, *22* (8), 3010–3013.
